# Comparison between acupotomy and corticosteroid injection for patients diagnosed with different classifications of tennis elbow: a randomized control trial

**DOI:** 10.1186/s13018-022-03323-x

**Published:** 2022-09-29

**Authors:** Lin-Pu Ge, Xiao-Qian Liu, Rui-Kun Zhang, Zhi-Neng Chen, Feng Cheng

**Affiliations:** 1 Department of Orthopedics, The Third Affiliated Hospital of Zhejiang Chinese Medical University, Hangzhou, 310005 Zhejiang China; 2 Zhejiang Chinese Medical University, Hangzhou, 310053 China; 3 Department of Endocrinology, Hangzhou Hospital of Traditional Chinese Medicine, Hangzhou, 310007 China

**Keywords:** Acupotomy, Corticosteroid, Tennis elbow, Classification, Single RCT, Alternative medicines

## Abstract

**Background:**

Tennis elbow has long been one of the most controversial subjects in orthopaedics. Many scholars thought the use of open or arthroscopic surgery was reserved for patients with refractory symptoms. Therapy with percutaneous acupotomy performed under local anaesthesia also removes degenerated tissue, releases strain, and therefore provides an alternative treatment option to surgical excision.

**Methods:**

The aim of this single-blinded randomized control trial was to examine the long-term clinical effectiveness of a nonsurgical percutaneous release technique (acupotomy) and the current recommended treatment (steroid injection) in people diagnosed with a refractory tennis elbow. Ninety patients with refractory symptoms were included. The intervention period was 6 weeks. According to the classification, 38 patients had extra-articular tennis elbow, 36 patients had intraarticular tennis elbow, and 16 patients had mixed type tennis elbow. Forty-five patients were randomly assigned to treatment with percutaneous release by acupotomy according to their classified condition, and 45 patients were randomly assigned to treatment with steroid injection alone. The visual analogue scale (VAS), a tenderness assessment, a grip assessment, and the Nirschl staging system were used for outcome evaluation at pretreatment and the posttreatment timepoints from 12 to 48 weeks.

**Results:**

During the first weeks, there were no differences observed between the groups. By 6, 24 and 48 weeks, significant differences were observed between the two groups. The acupotomy group scored significantly better in visual analogue scale score (VAS) of pain, tenderness during palpation, pain-free grip strength (PFGS) and Nirschl staging than the corticosteroid group.

**Conclusions:**

For patients with lateral epicondylitis, acupotomy is just as effective as corticosteroid injections in the short term (< 6 weeks). In the long term, acupotomy has greater efficacy and is associated with a lower rate of recurrence than corticosteroid injections in the management of lateral epicondylitis.

*Trial registration*: The National Health Commission announced the "ethical review measures for biomedical research involving people" in 2019, which was not mandatory in previous studies.

## Background

Lateral epicondylitis (LE), which is also known as “tennis elbow”, is a condition that comprises a myriad of symptoms that occur along the lateral aspect of the elbow; it is characterized by pain in the elbow and grip force reduction [1]. LE is a common source of elbow pain and is considered to be a degenerative tendon disease [2], and the condition exhibits an incidence of approximately 1–3% of the population and is usually noted in patients aged between 35 and 50 years [3]. It is a chronic tendinitis of the extensor muscles and primarily effects the extensor carpi radialis brevis [4]. LE was first described as a clinical entity by Runge in 1873 [5]. A variety of therapeutic interventions have been described in the literature, including wait-and-see, physiotherapy, corticosteroid injection, autologous blood injection, and surgery (percutaneous, arthroscopic or open), and even acupuncture and Botox infiltrations have been attempted with variable success rates [6–9]. All studies reported good but inconclusive results; thus, the treatment of LE remains controversial. Tonks et al. thought that steroid injection therapy was the best conservative treatment and that the symptoms of pain could be rapidly relieved during the initial phases of the condition [10]. Some experts suggested that corticosteroid injection should not be used to treat most patients with tennis elbow who exhibit a symptom duration of less than 12 months and that this approach might be less beneficial to the long-term outcomes of the patient [11]. In the long term, physiotherapy became the best option, followed by a wait-and-see policy, but the recovery period was still long [12]. While Houck et al. reported that autologous blood and platelet-rich plasma are effective treatments in the intermediate term [13]. Open surgery approaches mainly concentrated on releasing the origin of the extensor and offered long-term results [14]. The literature research showed favourable results for LE patients treated with arthroscopic surgery, patients reported to be satisfied with the operative results in terms of VAS, DASH score, time for returning to work, and overall outcomes, but the technical requirements were high, and the significant improvements were found on functional measures [15]. Cho, B. K.et al. reported that the mini-open surgical procedure was an effective method for LE [16]. Mattie, R.et al. described a replaceable treatment option for percutaneous tennis elbow release [17]. Percutaneous techniques have recently gained popularity and have a high rate of cure in patients [18]. However, currently, the literature does not support the superiority of any one technique [19]. Acupotomy, which utilizes a specially designed needle called a small needle knife and is a new nonsurgical percutaneous release technique for tennis elbow, has been widely used as a treatment for tendinopathy in China for more than 50 years and has gained popularity in recent years [20]. Acupotomy and acupuncture are traditional Chinese medicine treatments which can relieve the compression of local nerves and blood vessels. In addition to a satisfactory curative result, the best benefits of the percutaneous approach appear to be lower morbidity, a minimally invasive technique and earlier return to work and activity. Moreover, acupotomy allows repeated operation and does not affect subsequent treatment [21]. Although acupotomy was found to be an effective method for the treatment of tennis elbow, the therapeutic effect of acupotomy was not satisfactory according to routine clinical practice, especially for the treatment of refractory tennis elbow. Tennis elbow cannot be explained by a single pathogenic mechanism, and patients should be given different treatments according to the clinical manifestations, local manifestation, and the different types of tennis elbow.

The purpose of our study was to evaluate two different techniques for tennis elbow. Our hypothesis was that percutaneous release, a minimally invasive technique (acupotomy), would exhibit better long-term effectiveness in LE patients than corticosteroid injection alone with respect to pain relief, functional recovery and recurrence rate.

## Materials and methods

This prospective, randomized control study was performed between February 2013, and June 2014 at the Department of Orthopaedics of the Third Affiliated hospital of Zhejiang Chinese Medical University, Hangzhou, China. The prospective data included in this study were obtained as part of the routine care of the patients and were part of their medical records. All patients in this study were screened using the predetermined sealed-envelope method. A clinical diagnosis of LE was made on the basis of a history of lateral elbow pain that was triggered or exacerbated by wrist extension generally or during specific activities and point tenderness of the lateral epicondyle [22]. The duration of LE in each participant was more than 3 months, and the participants were over 18 years old. All patients underwent a standard radiographic examination before treatment, and no patients had been treated with injections during surgical intervention or other treatments that would affect the condition for 6 months. The exclusion criteria were a history of previous elbow injury, a history of surgery, the presence of coexisting neck or thorax pathology, the presence of a tumour, infection or ganglion in the region of the elbow, elbow arthritis, and a history of clotting disorder or diabetes mellitus. The procedures and aim of this trial was explained to the patient by an investigator who provided the procedures. The pain and functional recovery using a visual analogue scale (VAS), grip strength, and the Nirschl staging system for an initial period of 6 weeks after treatment and during a follow-up period of 48 weeks by a randomized, controlled, assessor-blinded clinical trial based on the classification of tennis elbow.

### Sample size calculation

The primary efficacy parameter was the change in Mid-Atlantic Shoulder and Elbow Society (MASES) scores from baseline to the end of treatment after 2 weeks. According to our preliminary test and a previous study [10, 20], a sample size of 45 patients per group provided 80% power to detect a clinically meaningful difference between group I and group II at an alpha level of 0.05 (2-tailed test). Approximately 39 participants in each group were calculated to be needed. In addition, to account for a dropout rate of 10%, at least 45 participants were enrolled in each group.

### Classification of tennis elbow

Extra-articular type tennis elbow was classified as pain that appeared in the lateral region of the elbow that was aggravated by working and that could be alleviated at rest. Additionally, in some of these participants, the tenderness was limited to the outside of the humerus condyle, elbow motion was unrestricted, Milton's character and wrist extensor strain test results were positive, and bone hyperplasia of the outside humerus condyle was present on the X-ray. Intraarticular type tennis elbow was defined as pain that appeared within the humerus oar joint during passive activities. In these patients, the elbow pain was more severe and could not be alleviated at rest. Tenderness was mainly limited to humerus oar joint spaces. A region of synovial hypertrophy could be observed, and elbow motion was restricted. Humerus oar joint hypertrophy of the synovial shadow could be observed on X-ray. Mixed type tennis elbow was the diagnosis when the above characteristics of both classifications or other symptoms were present.

### Treatment protocols and procedure

**Fig. 1:**
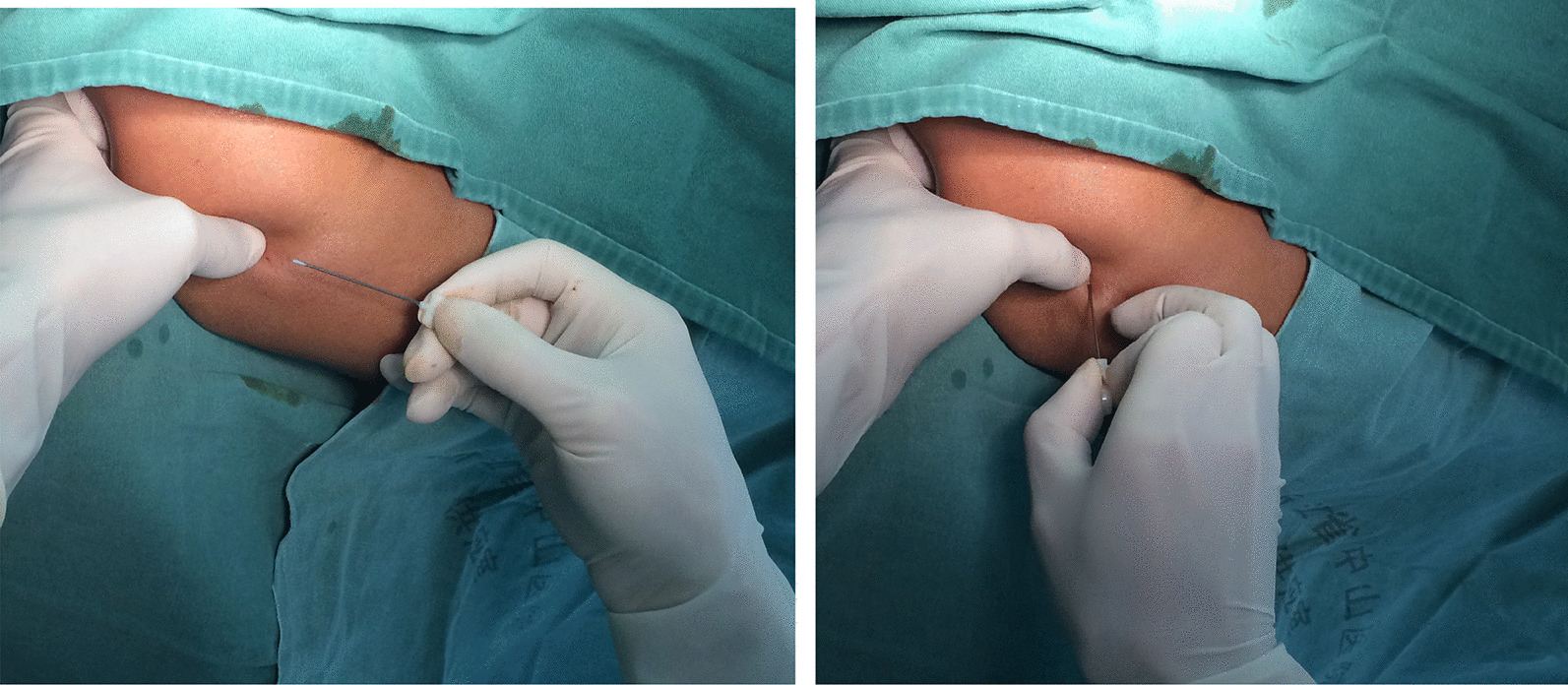
A clinical photograph of the specially designed needle and the acupotomy treatment at the lateral epicondyle

We treated LE patients using a specially designed needle (Hanzhang specially designed needle (Huaxia Meditech 53 Inc., Beijing, China)) that was 0.6 mm in diameter and 50 mm long. It was streamlined and solid. The tip was planus to facilitate its insertion into the skin. The width of the blade was 0.6 mm to release the tendinous tissue, which was safer and produced less damage (Fig. 1).

In the outpatient department, after confirmation of the clinical diagnosis, patients in group I were treated with a specially designed needle. The technique was as follows (Fig. 1). The point of pain was located at the lateral epicondyle of the humerus by palpation. The local area of the skin was cleaned, and 1% plain lignocaine was injected using a 25-gauge needle. For patients with extra-articular type tennis elbow, the specially designed needle was inserted through the skin at 90°, and the bevel of the needle was placed parallel to the long axis of the forearm muscle fibres, blood vessels, and nerves. The cutting and peeling technique was performed close to the bone and repeated twice. At the same time, an assistant documented upper limb flexion of elbow, flexion of the wrist, pronation and the activities of the elbow. For patients with intraarticular type tennis elbow, the specially designed needle was inserted through the skin at 90°, and the bevel of the needle was placed transverse to the humerus oar joint spaces. The transverse oscillation technique was performed along the joint space and repeated 3–4 times. At the same time, an assistant recorded upper limb flexion of the elbow, flexion of the wrist, pronation and activities of the elbow. For patients with mixed type tennis elbow, according to the specific signs, the above treatments were used and combined at the actual pressure point. Patients in group II were administered special injection therapy. The injection therapy consisted of a single injection of 1 mL of diprospan and 2% lignocaine hydrochloride made up to a volume of 2 mL, which was injected into the symptomatically tender region of the common extensor origin. The treatments of the two groups were administered once a week and three times total as a course of treatment.

### Clinical evaluation

Patients were asked to evaluate the overall pain using a visual analogue scale, and tenderness and grip strength assessments were used to evaluate changes in the symptoms. The Nirschl staging system was used to evaluate elbow function. With reference to Nirschl staging [23], the criteria used to assess the curative effect of tennis elbow treatment were divided into seven grades. Outcome assessments were performed by the investigator before randomization and 6 weeks, 12 weeks, 24 weeks and 48 weeks after the start of the treatment. Patients were asked to evaluate the pain using a visual analogue scale (VAS 0: no pain; VAS 10: extreme pain) at rest, during daily activities, and during sports or work in specific situations. Tenderness was recorded with an M-tone digital pressure tester (Tianjin Ming Tong Century Technology Co., Ltd.) before and after the treatment, which produced the stress of tenderness. The pain-free grip strength (PFGS) was measured in kilograms when patients gripped a load grip strength apparatus (cixi ZongHanYan shun Plastic hardware factory production) before and after treatment while patients were in a seated position. The patients were instructed to slowly squeeze the dynamometer and to stop the instant any discomfort was first felt. The results of three attempts separated by 1 min intervals were recorded, and the mean value in kilograms was calculated.

Elbow function was evaluated according to the Nirschl system, which consists of seven phases in ascending level of the severity of pain (Table 1). It ranges from phase 1 (mild pain with exercise, resolves within 24 h) to phase 7 (constant pain at rest, disrupts sleep).

**Table 1:** Details of the Nirschl staging system

Phase description
1 Mild pain with exercise, resolves within 24 h
2 Pain after exercise, exceeding 48 h
3 Exercise pain does not alter activity
4 Pain with exercise alters activity
5 Pain with heavy activities of daily living
6 Pain with light activities of daily living, intermittent pain at rest
7 Constant pain at rest disrupts sleep

### Statistical analysis

All data were analysed by a blinded statistician using the SPSS (Statistical Product and Service Solutions) statistical package program (version 17.0, SPSS Inc., Chicago, Illinois, United States) at a location that was different from the third clinical research institute of Zhejiang Provincial Hospital of Chinese medicine. Data were analysed using a paired samples *t* test and repeated-measures analysis of variance (ANOVA). All patients randomized to each group were included in the analysis, and the data analysis was conducted using two-sided significance tests at the 5% significance level. Quantitative data with continuous variables are expressed as the mean ± SD. Differences between groups were analysed with one-way ANOVA, and a *t* test was used for comparisons of two independent samples. Hierarchical data were ranked and assessed, with a *P* value of < 0.05 indicating that the difference was statistically significant.

## Results

A total of 90 patients were recruited for this study from February 2013, to June 2014. However, only 68 patients were included in this study; eight were excluded due to default, and fourteen were lost to follow-up. According to the classification system, 34 patients had extra-articular type tennis elbow, 21 patients had intra-articular type tennis elbow, and 13 patients had mixed type tennis elbow. The baseline characteristics of group I (acupotomy) and group II (corticosteroid injection) were showed in Table 2.

**Table 2:** Characteristics of group I (acupotomy) and group II (corticosteroid injection)

Characteristic	Group I	Group II	*p* value
	(*n* = 34)	(*n* = 34)	
*Sex*			
(Male/female)	18/16	13/21	0.330
*Age*			
(Years)	46.2 (19–65)	44.2 (21–63)	0.518
*Side*			
(Left/right)	21/13	20/14	1
*Duration of symptoms*			
(Months)	5.7 (3–10)	5.6 (3–12)	0.925
*Employment (n, %)*			
Manual	10 (29.4)	8 (23.5)	
Nonmanual	24 (70.6)	26 (76.5)	0.784
*Classification (n, %)*			
Extra-articular type	16 (47)	18 (53)	
Intra-articular type	11 (32)	10 (29)	
Mixed type	7 (21)	6 (18)	0.886

Preoperatively, the mean VAS scores, the level of tenderness observed during palpation, the mean PFGS values and the mean Nirschl stages were similar (*p* = 0.459, 0.518, 0.548 and 0.448) (Tables 3, 4, 5, 6).

**Table 3:** Mean visual analogue scale score (VAS) for pain at rest for group I (acupotomy) and group II (corticosteroid injection)

Follow-up	Mean (SD)VAS(Visual analogue scale) score (Pain at rest)		*t*	*p* value
	Group I	Group II		
Preoperation	6.8 (1.6)	6.4 (1.3)	0.884	0.459
1 week	3.1 (2.0)	3.0 (1.7)	2.096	0.744
6 weeks	0.9 (1.0)	1.5 (1.2)*	3.763	0.034
24 weeks	0.41 (1.0)	1.2 (1.1)*	2.186	0.002
48 weeks	0.35 (0.9)	1.1 (1.1)*	1.897	0.002

*means *p*<0.05

**Table 4:** Mean values of assessment of tenderness during palpation for group I (acupotomy) and group II **(**corticosteroid injection**)**

Follow-up	Mean (SD) tenderness (Pain at palpation, kg)		*t*	*p* value
	Group I	Group II		
Preoperation	1.80 (0.59)	1.89 (0.53)	0.307	0.518
1 week	3.53 (0.93)	3.39 (0.92)	0.103	0.549
6 weeks	4.81 (0.60)	4.50 (0.57)*	0.269	0.029
24 weeks	5.50 (0.55)	4.93 (0.54)*	0.338	< 0.0001
48 weeks	5.53 (0.53)	4.98 (0.50)*	0.963	< 0.0001

*means *p*<0.05

**Table 5:** Mean pain-free grip strength (PFGS) values for group I (acupotomy) and group II (corticosteroid injection**)**

Follow-up	Mean (SD) pain free grip strength (PFGS, kg)		*t*	*p* value
	Group I	Group II		
Preoperation	23.80 (3.90)	24.43 (4.74)	2.419	0.548
1 week	37.79 (5.00)	38.69 (4.39)	0.597	0.430
6 weeks	42.73 (5.01)	40.07 (4.15)*	3.318	0.020
24 weeks	46.28 (4.50)	42.48 (5.52)*	1.436	0.003
48 weeks	46.37 (4.21)	42.24 (5.51)*	3.143	0.001

*means *p*<0.05

**Table 6:** Mean Nirschl stage scores for group I (acupotomy) and group II (corticosteroid injection)

Follow-up	Mean (SD) Nirschl stage		*t*	*p* value
	Group I	Group II		
Preoperation	5.9 (1.0)	5.8 (1.0)	0.125	0.448
1 week	3.1 (1.5)	3.0 (0.9)	12.818	0.846
6 weeks	0.5 (0.9)	1.1 (1.1)*	2.813	0.018
24 weeks	0.41 (1.0)	1.2 (1.3)*	7.482	0.004
48 weeks	0.32 (0.7)	1.1 (1.2)*	22.862	0.003

*means *p*<0.05

**Fig. 2:**
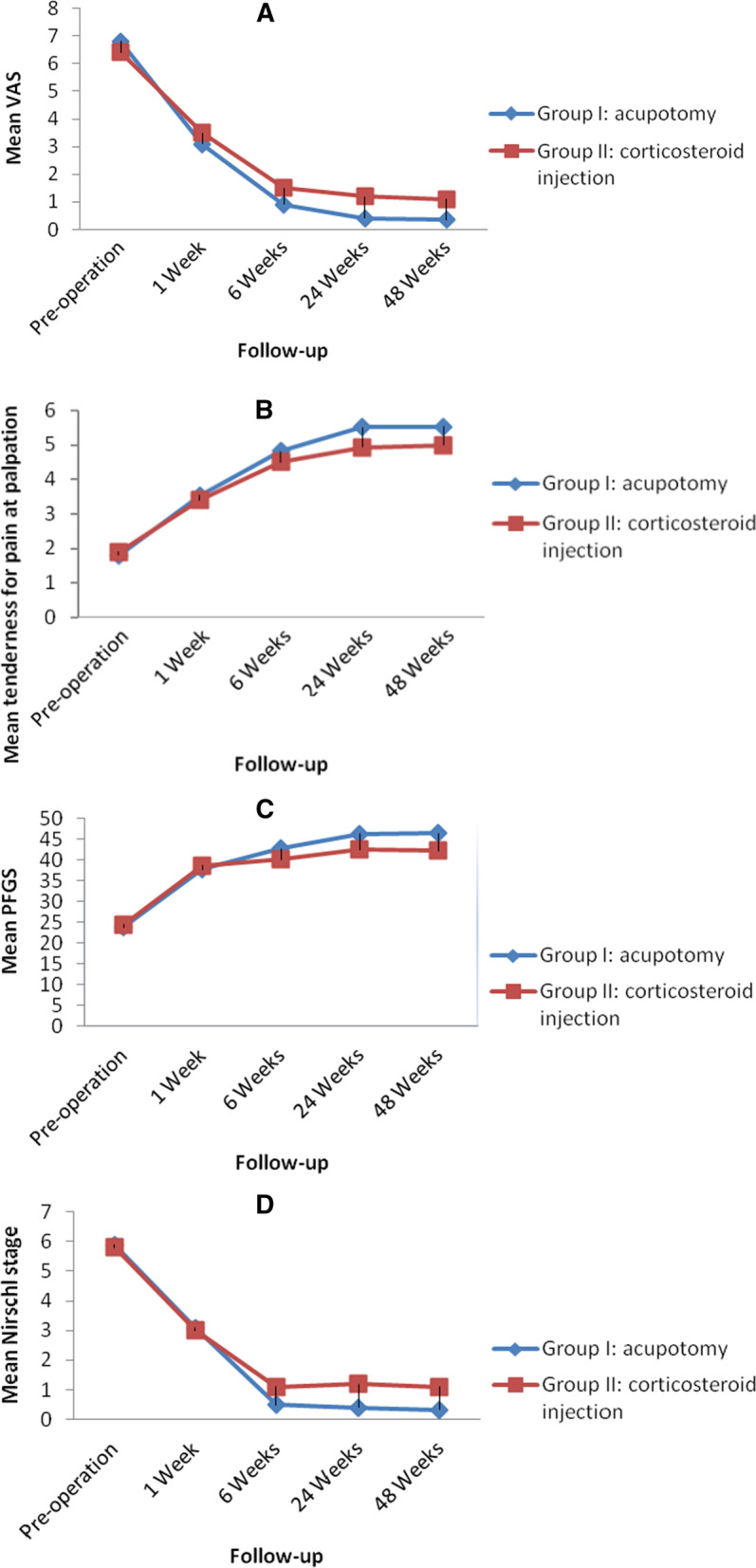
**A** mean visual analogue scale (VAS) of pain. **B**. mean tenderness during palpation. **C**. mean pain-free grip strength (PFGS). **D**. mean Nirschl staging

The results of the VAS for pain at rest, assessment of tenderness during palpation, and the PFGS and Nirschl grades followed a remarkably similar course over the follow-up period, as shown in Fig. 2A to D, respectively. Initially, there was no significant difference observed in the VAS for pain at rest, the tenderness during palpation, the PFGS and Nirschl scores between group I and group II at one week (*p* = 0.565, *p* = 0.549, *p* = 0.430 and *p* = 0.846, respectively). A statistically significant difference between two groups was found from 6 to 48 weeks. The mean VAS scores and mean Nirschl stages of two groups both decreased, while group I descended significantly compared to group II with increasing length of follow-up (*p* < 0.05). The Mean values of assessment of tenderness during palpation and the PFGS of two groups both increased, while greater grouth has been observed in group I with increasing length of follow-up (*p* < 0.05).

At the 24-week and 48-week follow-ups, a total of 28 patients (82%) in group I were completely relieved of pain, as were 16 patients (47%) at week 24 and 17 patients (50%) at week 48 in group II (*p* < 0.001, Chi-squared test). However, only 15 patients (44%) in group I and 11 patients (32%) in group II were pain-free at six weeks, but there was no recurrence by six months, which was a result that reached statistical significance (*p* < 0.001, Chi-squared test). This investigation suggests there were very few patients with intraarticular and mixed types of pain, and pain was completely relieved in group II at the 24- and 48-week follow-ups. Only three patients (8.8%) exhibited local skin atrophy in group II, while no patient in group I exhibited this problem, but this result did not reach statistical significance (*p* = 0.150, Chi-squared test), demonstrating that local steroid injection performed with proper care minimizes the occurrence of this complication. No patients reported elbow stiffness, infection, reflex sympathetic dystrophy, postinjection flare, facial flushing, neurovascular damage, tendon rupture or other untoward complications.

## Discussion

Although tennis elbow is a benign self-limiting condition that improves with or without treatment within 12 months [24], this amount of time is too long for patients to suffer from pain and disability. What patients often require is a safe and minimally invasive procedure that can enable them to return to their daily activities as soon as possible. The features of tennis elbow are microvascular damage, degenerative cellular processes, and disorganized healing [25]. Although the pathogenesis of tennis elbow remains unclear, the pathology of each type of tennis elbow is not all the same. Some researchers suggested that the existence of contact pressure between the origin of the common extensor tendon and the lateral side of the capitellum in fresh cadaveric upper extremities causes tennis elbow [26]. The pathophysiology was found to be more related to chronic changes in the degenerative tissue than to actual inflammation. The mechanism underlying the pain that occurs during lateral epicondylitis involves an inflammatory process, but biopsies from the common extensor origin and surrounding structures often do not show lymphocyte infiltration but do show vascular hyperplasia, disorganized collagen, and dense populations of fibroblasts [27]. High-resolution ultrasonography can help clinicians visualize key anatomic structures of the elbow and guide periarticular and intra-articular injections. Historically, most procedures done around the elbow have been done using landmark guidance, and few studies have reported the accuracy of ultrasonography-guided injections in the elbow region.

In Traditional Chinese Medicine, small needle-knife therapy is a technique that combines both acupuncture and micro-invasive surgery. It was introduced in China from 1976, and it uses a new-style bladed needle that has a flat head and a cylindrical body, primarily used to treat soft tissue injuries and bone hyperplasia [28]. According to the theory of acupotomy on chronic soft tissue injury, the tendon attached to lateral epicondyle of humerus causes compensatory self-repair and self-regulation after injury, forming local adhesion, scar and contracture. The small needle-knife will release the adhesion of the injured tendon, scrape off the scar, cut the neurovascular bundle, and restore the local dynamic balance to cure LE. In this study, we found that the patients who received acupotomy treatment didn't exhibit statistically significantly better values for all outcome measures at the 1-week follow-up than those who received steroid injection. There was no statistically significant difference between the two groups in any of the outcome measures at the 1-week follow-up. However, there were significant differences between the two groups at the 6-, 24- and 48-week follow-ups. This study demonstrated the beneficial effects of applying a nonsurgical percutaneous release technique on the pain, grip, tenderness and dysfunction that are classically associated with tennis elbow or LE. In particular, the curative effects of acupotomy treatment were significantly better for patients with the intraarticular and mixed types than those of steroid injection. This technique is based on the principle of classification advocated by Xinmiao Yao [20]. We realized that tennis elbow could not be explained by a single pathogenic mechanism. Tennis elbow patients should be classified according to the clinical symptoms and signs of parting, and patients with different types of tennis elbow should be given different treatments in clinical practice. According to the classification scheme, extra-articular type tennis elbow is diagnosed as epicondylitis, which is characteristic of terminal illness, periosteum tendinitis, and chronic aseptic inflammation of soft tissue. Its pathogenesis could be understood to occur as a result of repeated stretching of the wrist and elbow, and it could be caused by strain lesions located on the outside epicondyle of the humerus by the former arm extensor. The purposes of therapy for patients with this type of tennis elbow are to restore the normal stretch of the affected area and eliminate local chronic aseptic inflammation. An intraarticular type of tennis elbow is diagnosed as brachial radial joint lateral synovial bursa phlogistic and humerus oar joint osteoarthritis. The main pathologic change occurs inside the humerus oar joint or within an incarcerated humerus oar joint synovial membrane. The purposes of therapy for patients with this type of tennis elbow are to release the abnormal tension and relieve the incarcerated synovial humerus oar joint.

Some researchers found no inflammatory biomarkers in the extensor carpi radialis brevis tendon in patients with LE [29]. Some researchers demonstrated areas of hypovascularity in the lateral elbow and suggested that these areas may lack sufficient blood supply to generate a normal inflammatory cycle and a robust healing response [30]. Very few inflammatory cells were noted in these early tissue samples. There was little evidence that traditional therapies, such as ice, physiotherapy, or nonsteroidal anti-inflammatory drugs (NSAIDs), significantly improved outcomes. Corticosteroid injections were considered to be safe for trials in refractory cases of epicondylitis [10]. However, these approaches are still controversial. Some evidence has supported the idea that corticosteroid injections are successful in providing short-term pain relief that is better than that provided by other approaches [31]. Like corticosteroid injections, the acupotomy treatments were found to be very effective in providing short-term pain relief. Glucocorticoid injection could relieve temporary pain by eliminating inflammation and improving the blood supply by expanding blood vessels, which might weaken the wrist flexor or extensor tendons or further aggravate tendinosis and increase the amount of the degeneration of radiocapitellar cartilage that is associated with tennis elbow syndrome [32, 33]. Therefore, the effects of treatment were not lasting, and patients easily experienced relapse. According to different pathological mechanisms, the different treatment methods were administered using different acupotomy treatments. Therapy can restore the normal stretch of the affected area by releasing abnormal tension, improving the blood supply through inflammatory stimulation, relieving the incarcerated humerus oar joint synovial tissue and reconstruction of vascular fibroblast structure by cutting. Although both treatments relieved symptoms, our results showed that acupotomy treatments administered based on classification were more effective than injection of steroids in the treatment of LE for long-term effects. The possible mechanisms could involve the release of abnormal tension and contact pressure between the extensor origin and the lateral side of the capitellum and inflammatory stimulation to restore the local tissue. The results of this study might also imply that relieving this contact is the logical approach for use in other treatment or arthroscopic procedures in which the extensor origin is detached or debrided.

## Conclusion

Although both treatments relieve symptoms, our results have shown that acupotomy treatments were more effective than injection of steroids in the treatment of LE for producing long-term effects, especially in patients with intraarticular and mixed type LE.

## Data Availability

The data are included in the article as figures and tables. All data generated during this study are included in this published article.

## Abbreviations

MASES: Mid-Atlantic shoulder and elbow society
LE: Lateral epicondylitis
VAS: Lateral epicondylitis
PFGS: Pain-free grip strength
SPSS: Statistical product and service solutions
ANOVA: One-way analysis of variance
